# Supplementary material for: Are carbon emissions trading and green financial instruments synergistic? -Comprehensive quantitative research based on content analysis
Source: PLoS One. 2024 Mar 7;19(3):e0298601. doi: 10.1371/journal.pone.0298601 (PMC10919601; doi:10.1371/journal.pone.0298601)
Supplement: S1 Data — (ZIP) [file pone.0298601.s001.zip › Data/地级市Z_F工作报告.zip!/║╙─╧/┬σ╤⌠╩╨/└┤╫╘╣1⁄2╓┌║┼í╛┬φ┐╦╩2╛▌═°í┐_2005.docx]

各位代表：

　　现在，我代表市人民政府，向大会作工作报告，请予审议，并请各位政协委员和列席人士提出意见。

　　一、2004年工作回顾

　　2004年是我市在全面建设小康社会进程中迈出重要步伐的一年。一年来，市人民政府在省委、省政府和市委领导下，在市人大、市政协监督支持下，牢固树立科学发展观，努力在发展中顺应调控，在调控中寻求机遇，迎难而上，奋发而为，圆满完成了市十二届人大一次会议确定的各项任务。

　　——经济总量跃上新台阶。全市生产总值达到904.6亿元，财政一般预算收入完成42.8亿元(按所得税改革前老口径完成50.6亿元)，分别比上年增长15.9%和36.1%，是2000年的2.1倍和2.4倍，提前实现了比2000年翻一番的目标。限额以上工业销售收入、全社会固定资产投资、社会消费品零售总额等主要经济指标也同步实现了翻一番。

　　——三次产业协调发展。完成第一产业增加值87亿元，增长6%，粮食总产208万吨，是历史上第二个丰收年；第二产业增加值533亿元，增长19%，其中限额以上工业增加值256.3亿元，增长31.8%，对GDP增长的贡献率达50%；第三产业增加值284.6亿元，增长13.9%。

　　——投资、消费、出口全面拉动。全社会固定资产投资完成372.6亿元，增长46.8%。社会消费品零售总额达到288.8亿元，增长16.2%。外贸进出口总额7.5亿美元，增长68%，其中出口5.9亿美元，增长79.4%。

　　——企业效益、财政收入、居民收入同步增长。限额以上工业实现销售收入771亿元，增长36%；利税总额74.3亿元，增长41%；利润33.3亿元，增长74%；综合效益指数达到147，同比提高27个百分点，创历史最高水平。全部财政收入91.4亿元，增长32.6%。城市居民人均可支配收入9032元，增长12.8%。农民人均纯收入2585元，增长13.5%。金融机构存贷款余额分别达788.9亿元和494.5亿元，比年初增加102亿元和74.3亿元，其中城乡居民储蓄存款余额518.8亿元，比年初增加66亿元。

　　这些成就，标志着我市经济社会发展跃上了一个新的战略起点，特别是主要经济指标提前实现了比2000年翻一番，这是我市在本世纪头四年取得的辉煌成就，是全面建设小康社会征程上的一个重要里程碑，为早日实现第二个翻番奠定了坚实基础。

　　(一)加快结构调整步伐，新的经济增长点不断涌现

　　工业四大产业基地初具规模。全年实施千万元以上工业项目168个，其中竣工项目75个；总投资361亿元，当年完成工业投资140亿元，超过了前三年的总和。在国家实行宏观调控的新形势下，我市仍有一大批项目争取到国家、省批准，并获国债和省结构调整资金支持。其中一拖年产8万台100/105系列柴油机、洛铜10万吨高精度电子铜板带、中硅公司300吨多晶硅、中信重机大型采选煤设备、洛玻超薄玻璃等9个项目被国家发改委列入国债支持计划，总投资27.8亿元；洛耐高性能硅砖、轴研科技精密轴承等11个项目列入省工业结构调整和高新技术产业化专项资金计划，获省、市结构调整和高新技术产业化贴息4000万元。全年先进制造业基地共有一拖推土机技改、中信重机大型水泥设备、联众纺织5万锭精梳纱等19个项目竣工。与此同时，华润2×60万KW机组、双源2×30万KW机组、新区2×13.5万KW机组等项目得到国务院批准并开工建设，在建电源项目总投资达到155亿元，总装机容量378万KW，全部建成后我市火电装机总容量将达630万KW以上。以伊川电力20万吨铝板带、新安电力15万吨铝板带和2万吨铝箔、首龙集团1万吨铝箔等项目为标志，总投资达110亿元的铝工业项目总体进展顺利，部分项目已投入试生产。洛阳石化2万吨双向拉膜、黎明化工院10万吨双氧水等8个石化工业项目已经竣工，18万吨聚酯、15万吨短丝等13个项目正在实施，800万吨炼油扩能、45万吨PX、60万吨PTA等项目已得到中石化批准，前期工作进展顺利。科研院所高新技术产业化步伐加快，原部属的12家科研院所完成技工贸总收入62亿元，同比增长72%。

　　农业六大支柱产业不断壮大。优质粮食、畜牧养殖、中药材、烟叶等农业产业化基地初具规模，六大支柱产业占第一产业总产值的69.6%。烟叶收购量首次位居全省第一。奶牛存栏4.3万头。各类农业产业化组织达487个，实现销售收入16亿元，带动农户55万户。小城镇带动能力逐步增强，工商税收同比增长21.2%。全市城镇化率达到36%左右。

　　旅游业蓬勃发展。投入4.6亿元加强旅游景点基础设施建设，景区品位进一步提升。旅游宣传促销力度加大，龙门石窟、白云山、鸡冠洞等景点在全国知名度不断提高。牡丹花会、河洛文化旅游节、中原滑雪节等节庆活动丰富多彩，“五一”、“十一”黄金周接待人数再创新高。全年接待国内游客1785万人次，境外游客9万人次，旅游总收入82亿元，分别增长25.5%、236%和26%。

　　消费品市场繁荣活跃。13个投资上千万元的大型市场项目进展顺利，润峰广场、王府井百货、丹尼斯二期等建成开业，百年家居城一期完工。居民消费结构明显升级，住房、汽车、通讯、电脑等成为新的消费热点。体育健身、文化娱乐、卫生保健和社区服务等消费快速增长。成功举办了首届国际鞋业交易博览会。

　　非公有制经济发展势头强劲。坚持中小企业改制转化一批、招商引资催生一批、经济能人领办一批、现有企业做大做强一批等有效途径，强力推进非公有制企业骨干带动工程，新上千万元以上非公有制项目290个，总投资126亿元，当年完成投资69.7亿元。全年非公有制企业实现营业收入800亿元，税收30亿元，分别增长36%和47%，营业收入超亿元企业由上年的23家增加到53家。非公有制经济占经济总量的比重达到41%左右。

　　(二)狠抓重点项目建设，经济社会发展支撑能力显著增强

　　努力争取国家和省投资，新列入国债计划项目58个(不含工业项目)，到位国债及省补助资金5.2亿元。加强银企合作，新增工业项目和流资贷款44.5亿元。利用市建设投资公司、优势企业等平台，多方筹资40多亿元。全年城镇以上投资完成270亿元，增长51.9%。

　　洛阳新区开发取得阶段性成果。全年开工各类工程474项，完成投资99.9亿元。开元大道西延长线、太康路等24条道路及配套管网全部贯通，瀛州路、大学路等10条道路正在施工。房屋建筑工程开工320万平方米，其中43栋80万平方米的高层建筑主体封顶，127栋71万平方米的多层建筑竣工投用。市委、市人大、市政协迁入新区办公，1.2万名村民入住村民安置小区，新区中小学如期开学，新区水系初步形成，人防综合楼、商行办公楼等正在紧张施工。新区城市功能已经启动，城市轮廓初步显现。

　　城区改造成效显著。凯旋西路、定鼎北路和涧河治理等16项市政工程如期竣工。王城大道主干道交付使用，洛河大桥和陇海铁路大桥正在建设。瀍东污水处理厂进展顺利。广州市场步行街等城区改造项目已经完成，西工商业步行街、王城大道沿线改造等地块开发项目有序推进。房地产开发完成投资31亿元，商品房竣工202万平方米，销售168万平方米。全年新增城市道路82公里，新增绿地134万平方米，城市规模、城市形象发生了显著变化。荣获“中国十大最佳魅力城市”称号。

　　交通建设全面推进。全年完成交通投资25亿元。少洛、济洛、西南环城3条高速路基工程已经完成，其中济洛高速洛阳东出口段已经通车，实现了连霍高速与洛界高速的连接。临木路、西秋路等9条干线公路建成通车，连接市区与南部山区450公里的公路环线全部贯通。新建、改建县乡公路303公里，乡村汽车站142个，进一步方便了农村群众出行。

　　农村生产生活条件不断改善。全年完成各项农村基础设施建设投资16.9亿元。西霞院水库、禹门河反调节水库建设进展顺利，陆浑、段家沟、大沟口、玉马水库除险加固和陆浑西干渠、龙脖灌区、新安提黄灌区改造等项目有序实施。新增有效灌溉面积3.5万亩，治理土地9万亩。退耕还林22.2万亩，通道绿化169公里，上清宫森林公园基本建成。投资2.8亿元，完成491个行政村1567公里的“村村通”工程。投资1890万元，完成81所乡镇卫生院硬件建设和617名医务人员培训。4年来累计投入4.04亿元，在全省提前两年率先完成农村中小学D级危房改造任务，其中2004年投入1.85亿元，新建校舍55万平方米。全年安排各类扶贫资金1.49亿元，扶贫开发力度不断加大，移民式扶贫有效实施。

　　(三)各项改革稳步推进，对外开放成效明显

　　国有企业改革步伐加快。安龙、骏马、春都等企业资产重组成果进一步巩固，明花集团、海虹轮胎、制冷机械厂、744厂等重点困难企业改革取得突破。国有大中型企业主辅分离和分离企业办社会取得新进展，洛轴主业由永煤集团成功重组，一拖下属的9户辅业单位完成分离改制，石化总厂、洛铜、单晶硅、铁路分局等7户企业的20所中小学完成分离。中小企业改制积极推进，438户企业实施了改制措施，处置、盘活各类资产20.5亿元，安置职工2.4万人。加强钼、煤和铝矾土资源整合，资源依托型重点企业的改制正在规范。轴研科技、中色科技、中航光电等企业改制上市步伐加快。财政管理体制改革不断深化，部门预算、收支两条线、国库集中支付、政府采购等工作进一步加强。认真落实粮食直补、降低农业税税率政策，使全市农民减负增收9618万元。粮食收购市场和价格全面放开，国有粮食购销企业产权制度改革基本完成。农村信用社改革全面启动，清产核资和增资扩股任务已经完成，清收不良贷款工作有序推进。投资体制改革稳步实施，投资主体多元化趋势不断增强。政府机构改革基本完成，事业单位人事制度改革稳步推进。

　　认真落实“三个三分之一”工作法，广泛开展招商引资，成功举办了第22届牡丹花会。全年签订招商引资合同378个，开工项目320个，实际到位资金43亿元。新批外资企业46家，合同利用外资2.9亿美元；实际利用外资8900万美元，增长42%。签订对外工程承包和劳务合作合同9650万美元，完成营业收入3758万美元。

　　(四)加强社会保障和就业工作，切实解决群众实际问题

　　“两个确保”全面落实，按时足额发放离退休人员养老金10.9亿元、下岗职工基本生活费7190万元。认真落实提高养老待遇政策，企业退休人员人均月增49元。企业养老保险扩面3.3万人，清欠1.5亿元，综合征缴率达117%，实征养老金同比增加1.9亿元。失业、医疗、工伤保险征缴率分别达122%、101%和107%。全年累计享受最低生活保障人员118万人次，发放低保金7398万元，基本实现了应保尽保。认真落实就业再就业扶持政策，城镇新增就业10万人，实现再就业4.9万人，其中“4050”人员1.9万人。强力实施“农村百万劳动力转移工程”，完成技能培训13.9万人，输出农村劳动力73万人，劳务创收25亿元。加强农村贫弱群体基本生活保障，下拨救灾救济款2248万元，救助灾民36.4万人次，资助灾民重建房屋1.3万间，对6.5万农村特困人口实施了社会救助。全年清理建筑领域拖欠工程款和农民工工资5.65亿元。

　　(五)政府职能转变加快，经济发展环境进一步优化

　　认真贯彻行政许可法，积极开展行政许可事项清理，大力推进政务公开，取消行政许可项目28项。43个部门保留的195个行政许可项目、75个收费项目进入行政服务中心，实行“一站式审批、一条龙服务”。对377个单位集中开展了收支两条线专项检查和小金库清理工作，查处违纪资金8396万元，已收缴国库和财政专户3200万元。市长便民电话受理来电来信8352个，办结率97.7%。深入开展民主评议行风活动，对教育乱收费、公路“三乱”和医药购销、医疗服务中的不正之风进行了专项治理。积极开展食品、药品、农资、劣质钢材等整治活动，市场经济秩序进一步规范。健全安全生产管理机构，严格事故查处和责任追究，全面开展煤矿、非煤矿山、消防、交通等安全生产专项整治，各类安全事故数量同比下降53.6%，直接经济损失下降28.7%。

　　(六)科技教育和各项社会事业全面进步，精神文明和民主法制建设不断加强

　　科教兴洛战略得到较好实施。加大科技投入，积极争取国家、省科技项目，列入国家、省科技计划项目168项，省高新技术产业化贴息项目6项，共获资金支持2085万元，其中有色院、矿研院、洛玻的3个项目列入国家“863计划”。积极用现代信息技术改造提升传统产业，国家级制造业信息化示范城市建设进展顺利，对一拖、洛铜、白马等40户企业实施了以提高数字化水平为重点的制造业信息化重点项目。省政府和科技部共建洛阳装备制造业基地的方案得到批准。科研院所体制改革不断深化，科技成果就地转化步伐加快。全年完成市级以上科研成果248项，技术合同交易额2.8亿元，科技对经济增长的贡献率达到43%。高新区引进超千万元工业项目15个，总投资9.5亿元；实现技工贸总收入98.2亿元，同比增长22%。教育“两基”成果不断巩固，高中阶段教育得到加强，普通高招再创历史新高，职业教育和成人教育健康发展。高等教育招生规模不断扩大，河南科技大学和工业高专新校区如期接纳新生。

　　各项社会事业加快发展。积极推进文化建设，大型舞蹈诗《河洛风》成功演出，青年宫文化中心主体完工，群艺馆已投入使用，新闻出版、文化娱乐市场不断规范。城乡医疗服务体系进一步完善，卫生监督中心、疾病预防控制中心和传染病医院开工建设，新型农村合作医疗试点工作稳步开展，顺利通过省级卫生城市复核。积极创建国家环保模范城市，市区环境空气质量良好天数达156天，已经退出全国十大污染城市之列。社区管理和服务水平不断提高，第三批“星光计划”项目全部建成。加强城市规划管理，合理开发利用水、土地、矿产资源。实行最严格的耕地保护制度，新增耕地4万亩。计划生育圆满完成省下达的人口控制目标。全民健身活动蓬勃开展。统计对经济运行的监测预测水平明显提高。审计的经济监督职能不断增强。文物保护、民族宗教、外事、侨务、人防、气象、地方志等工作都取得了新的成绩。

　　精神文明和民主法制建设取得新成效。深入学习贯彻“三个代表”重要思想，广泛开展向许振超、任长霞、杨岳等先进典型学习活动。积极推进未成年人思想道德建设，集中开展了校园周边环境专项整治。大力开展“道德规范进万家、诚实守信万人行”等精神文明创建活动，创建文明景区、文明城市工作取得新成效。村务公开和基层民主管理工作进一步规范。加强“双拥模范城”建设，驻洛解放军和武警部队在维护社会稳定、支持地方经济建设中做出了重要贡献。加强法制宣传教育，坚持依法行政，努力建设法治政府，提请市人大审议通过法规4件，颁布行政规章5件。严格执行人大及其常委会通过的法规、决定和决议，自觉接受人大工作监督、法律监督和政协民主监督，人大代表议案、建议和政协委员提案全部办结。廉政建设和反腐败斗争取得新成效。深入开展“万名干部下基层、化解矛盾保稳定”活动，着力化解信访积案。加强社会治安综合治理，完善治安防控体系，严打整治成果进一步巩固，荣获全省综治先进市称号。强化公安基层基础工作，市区18个派出所建设全部完成，新建农村派出所12个、修缮改建17个。

　　各位代表，这些成绩的取得，是省委、省政府和市委正确领导的结果，是人大、政协积极支持的结果，是全市人民共同奋斗的结果。在此，我代表市人民政府，向全市广大工人、农民、知识分子、干部，向人大代表和政协委员，向驻洛部队指战员、武警官兵和公安干警，向各民主党派、工商联、人民团体和离退休老同志，向所有关心支持洛阳发展的各界人士，致以崇高的敬意和衷心的感谢！

　　肯定成绩的同时，我们也清醒地看到，经济社会发展中仍存在不少矛盾和问题：农业生产基础还不牢固，农业增效和农民增收的长效机制尚未形成；国有大中型企业体制转换相对缓慢，深层次矛盾依然存在；服务业发展相对滞后，旅游宣传促销工作有待进一步加强；招商引资中科技含量高、带动能力强的大项目偏少，实际利用外资总量与洛阳发展水平不相称；信贷资金和煤电油气运依然紧张，资源和环境约束日益明显；安全生产上还存在一些薄弱环节，社会治安状况仍不容乐观；就业再就业和社会保障压力加大；经济发展环境仍需优化，政府机关的服务意识和办事效率有待进一步提高。对此，我们一定高度重视，在今后工作中认真加以解决。

　　二、2005年的奋斗目标和工作重点

　　今年是我市经济发展至关重要的一年，我们不仅要确保实现生产总值超千亿、全部财政收入超百亿的宏伟目标，实现经济发展的历史性突破，同时还要为生产总值、财政收入等主要经济指标提前实现第二个翻番奠定坚实基础。综合各方面情况判断，今年我们面临许多加快发展的有利条件。从宏观形势看，我国经济仍处在新一轮增长周期的上升阶段，国家进一步加强和改善宏观调控，实行稳健的财政政策和货币政策，有利于保持经济平稳较快增长。从我市情况看，经过近几年的结构调整，四大产业基地迅速壮大，工业优势不断强化，综合经济实力明显提升，经济增长的自主机制逐步形成，快速发展的基础进一步增强。特别是在落实科学发展观和宏观调控政策过程中，我市牢牢抓住符合国家产业政策和洛阳实际的项目，抢抓机遇，顺势而为，一大批先进制造业、能源、石化、铝深加工项目获国家或省批准，为今后的发展提供了有力的项目支撑。同时，经过多年的交通扶贫和农村基础设施建设，农村生产生活条件发生了巨大变化，为加快农村经济发展奠定了良好基础。面对新的形势，只要我们在继续强化投资拉动的同时，积极培育消费拉动，努力扩大出口拉动，认真解决好“三农”问题，经济增长的潜力就会进一步迸发出来，不仅“千百亿”目标完全可以实现，而且还会发展得更快更好一些。

　　今年政府工作的总体要求是：以“三个代表”重要思想为指导，认真贯彻党的十六届三中、四中全会精神，坚持以科学发展观统领经济社会发展全局，坚定不移地实施“工业强市、旅游强市”战略，着力推进改革开放，强化投资和消费拉动，加快经济结构调整，转变经济增长方式，高度重视“三农”问题，大力推进工业化、城镇化和农业现代化，改善城乡居民生活，繁荣各项社会事业，积极构建和谐社会，促进经济社会全面协调可持续发展。

　　经济和社会发展的主要预期目标是：生产总值增长13%，全社会固定资产投资增长25%，财政一般预算收入增长11%，社会消费品零售总额增长15%，城镇居民人均可支配收入增长7.8%，农民人均纯收入增长6%，城镇登记失业率控制在4%以内，人口自然增长率控制在6‰以内。

　　完成上述目标，要重点抓好以下十项工作:

　　(一)加强“三农”工作，保持农业和农村发展的好势头

　　坚持把解决“三农”问题作为各项工作的重中之重，巩固提高粮食综合生产能力，加快农业结构调整，加强农村基础设施建设，千方百计增加农民收入，促进农村经济社会全面发展。

　　加大政策扶持力度。认真落实各项“三农”政策，保证行之有效的支农政策落到实处，已经给农民的实惠不减少。全面落实免征农业税政策，进一步减轻农民负担；巩固粮食流通体制改革成果，继续落实对种粮农民直接补贴、良种补贴和农机具购置补贴。进一步整合各项支农资金，集中支持农村基础设施建设和农业结构调整。加强农民负担专项治理和监督检查，防止农民负担反弹。

　　全面推进农村经济结构调整。围绕六大支柱产业，不断壮大农业产业化基地。加强基本农田保护，巩固粮食种植面积，努力提高粮食单产，力争优质小麦和优质玉米分别达到150万亩和85万亩，确保正常年景粮食总产200万吨以上。切实抓好奶业生产，大力发展规模化奶牛养殖。强化奶业企业资源整合，加强对外合资合作，努力做大做强，提高抵御市场风险能力。积极扩大烟叶种植面积，不断提高烟叶的质量和档次。商品牡丹、中药材、林果等产业都要注重提高质量和效益，大力培育产业优势。认真落实对龙头企业的各项扶持政策，培育壮大农产品加工企业。加快农产品的标准、质量认证和检验检测体系建设，扩大农产品绿色安全无害化生产。积极发展农村专业合作组织、农民经纪人队伍。大力发展劳务经济，加强组织协调和技能培训，力争完成劳动力转移培训20万人，劳务输出85万人，劳务创收30亿元。进一步提高小城镇辐射带动能力，多方招商引资，发展特色经济，培植财源，凝聚人气，力争引进千万元以上项目50个，新增小城镇常住人口5万人。

　　进一步改善农村生产生活条件。加快重点水利项目和灌区配套建设，着力增加有效灌溉面积。积极服务西霞院水库建设，按时完成移民搬迁任务。投资1.2亿元，抓紧开工建设孟西灌区。继续抓好新安提黄、陆浑西干渠、龙脖灌区配套工程，新发展有效灌溉面积3万亩、节水灌溉面积3万亩。投资2000万元，搞好玉马水库除险加固；投资5000万元，搞好第四批电气化县建设，完成禹门河、金牛岭等工程年度建设任务；投资1800万元，抓好农村饮水安全工程，解决5万人安全饮水。完成农业综合开发投资4620万元，治理土地5万亩。搞好以小流域治理为重点的水土保持工作，治理水土流失200平方公里。巩固退耕还林成果，完成退耕还林和荒山绿化25万亩。投资2.7亿元，完成439个行政村1285公里的“村村通”工程。投资715万元，完成35所乡镇卫生院建设和500名人员培训，确保今年全面完成乡镇卫生院建设。

　　(二)大力实施工业强市战略，加快结构调整和企业改革

　　按照走新型工业化道路的要求，坚持用高新技术和先进适用技术改造提升传统产业，着力转变经济增长方式。进一步发展壮大“四大产业基地”，认真抓好在建的和国家、省已经批准的项目建设，确保工业投资完成180亿元以上、限额以上工业销售收入突破1000亿元。

　　今年全市计划安排千万元以上工业项目187个，总投资470亿元，全部完成后可年新增销售收入700亿元以上。其中，续建项目109个，当年完成投资146亿元；新开工项目78个，当年完成投资51亿元。先进制造业基地方面，要确保一拖1万台重卡、中硅公司300吨多晶硅、中信重机矿井提升机等39个项目竣工投产，新开工洛铜10万吨高精度电子铜板带、一拖4万台重型柴油机、洛玻搬迁改造和超薄玻璃二线等30个项目。火电基地方面，要确保华润2×60万KW机组等项目完成年度施工任务，双源2×30万KW机组、新区2×13.5万KW机组等项目年底前基本投产，力争新增装机容量217万KW；继续抓好正村煤矿、何庄煤矿、义煤集团孟津煤矿等煤炭项目建设。铝工业基地方面，要确保伊川电力20万吨铝板带、新安电力15万吨铝板带和2万吨铝箔、首龙集团1万吨铝箔等项目如期投产，完成新安120万吨氧化铝工程年度建设任务。石化基地方面，洛阳石化18万吨聚酯、15万吨短丝项目7月份务必投产，800万吨炼油扩能、45万吨PX、60万吨PTA等项目要抓紧开工准备，力争早日开工。

　　加强工业经济运行的监测和协调，搞好电力、煤炭供需衔接和运力调配，确保工业生产特别是重点企业的正常运行。加强企业内部管理，大力推进体制创新、技术创新和管理创新，健全激励和约束机制，进一步挖潜增效，不断增强企业活力和竞争力，努力提高经济运行的质量和效益。

　　强化国有资产监督管理，认真履行国有出资人职责，推动企业建立规范的法人治理结构。紧紧抓住产权制度改革和大型企业主辅分离两个重点，支持国有大中型企业开展招商引资，引进战略投资者，吸纳民间资本，大力发展混合型经济，争取绝大多数国有工业企业实现股权多元化，年底前基本完成市出资国有大中型企业主辅分离、辅业改制和分离企业办学校任务。积极利用国内外资本市场，推动符合条件的国有企业规范上市。年内全面完成洛钢、中昊、春都、明花等重点困难企业的产权制度改革。继续推进中小企业改革，重点做好破产清算、资产处置、职工安置等工作。全面推开商贸流通、建筑交通和公用事业等领域的中小企业改制工作。支持各类中小企业的改革和发展，安排中小企业改革专项资金，将市级中小企业担保资金增加到1000万元。

　　(三)坚持旅游强市战略，努力扩大第三产业规模

　　高度重视第三产业的发展壮大，进一步优化产业结构，增强消费对经济增长的拉动作用。

　　努力实现旅游业的新突破。加大旅游宣传促销力度，积极与著名媒体、网站开展合作，努力打造洛阳“历史文化之都”和“休闲度假之都”的旅游整体形象。培育名牌旅游景区，着力建设龙门石窟旅游度假区、白马寺佛教文化区、伏牛山生态度假区、小浪底旅游度假区等十大旅游精品。办好第23届牡丹花会、河洛文化旅游节、关林国际朝圣大典、中原滑雪节等节庆活动。加快培育旅游骨干企业，支持有实力的旅行社做大做强。引导旅行社和旅游景点、宾馆饭店建立互惠互利机制，鼓励外地优秀旅行社进入洛阳旅游市场。改善旅游交通条件，不断增加航空班次、旅游专列和观光大巴。加强行业管理，完善旅游投诉的快速处理体系。加强旅游配套设施建设，提高接待和服务水平，力争台塑集团在洛投资的五星级酒店花会前开业，四星级老友谊宾馆年底前建成，空空导弹研究院在新区投资的五星级酒店年内开工建设。力争全市旅行社达到100家，星级饭店达到50家，接待境内外游客1900万人次，旅游总收入超过100亿元，主要景区门票收入超过2.5亿元。

　　积极扩大消费需求。完善促进服务业发展的政策措施，努力提高服务业在国民经济中的比重。进一步加快大型市场建设，全年力争完成投资15亿元。重点抓好中原康城、珠江路商业街等10个投资上亿元的项目，不断增强对周边地区的辐射能力。积极发展新型业态，大力推广现代营销方式，加快城市农贸市场改超市步伐，促进传统商业企业改造升级。完善社区服务设施，推动社区服务业快速发展。加强农村商业网点建设，鼓励城市商业向农村延伸，积极发展连锁超市、便利店等零售业态，努力扩大农村消费。深化住房制度改革，全面放开搞活二级市场，规范发展物业管理。加快房改房发证速度，为住房进入市场交易创造条件。扩大服务业的对外开放，积极发展信息、咨询、法律等新兴服务业。进一步完善消费政策，改善消费环境，培育消费热点，巩固和扩大消费需求。

　　(四)保持固定资产投资稳定增长，进一步完善基础设施

　　坚持投资拉动，努力争取国家和省资金支持，积极实施一批事关经济社会发展全局的重大建设项目，力争完成全社会固定资产投资470亿元。

　　加快洛阳新区建设。全年预计投资规模在76亿元以上，努力实现新区成规模、聚人气、出形象。加快开元大道以南开发建设，进一步完善市政基础设施，牡丹大道、大学路等17条道路，自来水加压站、污水处理厂、供热管网等13项公用设施年内建成投用；抓好道路绿化工程，新增绿地180万平方米；新建通水干渠23公里，形成水面1400亩。加快房屋建筑工程进度，体育馆、游泳馆花会前建成；世纪大厦、公务员小区、高层次人才小区5月份交付使用；商行综合楼、移动通讯楼、洛龙区行政综合楼等高层建筑年内全部投入使用；抓紧开工建设河南科技大学、工业高专二期等18个项目。妥善安置新区村民生产生活，8个村民安置小区全部建成入住。高度重视新区招商引资工作，加快土地出售和资金回笼，千方百计拓宽融资渠道；积极引进高新技术产业，大力培育第三产业，强化产业支撑，增强新区的生机和活力。

　　抓好老市区的改造和管理。全年力争完成投资64亿元。加快王城大道洛河大桥和陇海铁路大桥施工进度，确保花会前全线通车。继续抓好第六批“两创”工程，抓紧开工纱厂东西路、唐宫东路、天津路、太原路以及53条社区道路改造等市政工程，力争年内全部完工。已经规划的59个地块的开发改造，年内全部开工建设。全年商品房施工面积力争达到500万平方米，竣工200万平方米。坚持将商业网点建设和旧城改造结合起来，进一步提升建筑品位。深化市政、公用体制改革，维护城市规划的严肃性和权威性。加大对占道经营、马路市场、车辆乱停乱放的治理力度，不断提高城市管理水平。

　　全面加快交通建设。全年力争完成投资25亿元以上。确保济洛、少洛、西南环城3条高速公路9月底建成通车；开工建设太澳高速汝阳大安至汝州寄料段；积极创造条件，争取开工五县环新安—宜阳—伊川高速公路。全面完成洛宁长水至卢氏界、新安县城至新渑交界等4个续建项目，新增干线公路通车里程133公里；开工建设栾川秋扒至县城、栾川方村至卢氏界等8条160公里的干线公路项目，花会前完成连霍高速洛阳西出口连接线的拓宽改造，实现与王城大道全线贯通；完成洛宁下峪至故县段、郑三线孟津境内段等4条140公里干线公路项目的前期准备。积极配合郑州至西安高速铁路客运专线洛阳段建设。

　　(五)加快对外开放步伐，大力发展非公有制经济

　　努力提高对外开放水平。大力实施开放带动战略，坚持对外对内开放并举，不断创新招商方式，努力提高招商引资实效性。组织一批投资大、科技含量高的项目对外推介，力争国有大中型企业和科研院所在对外合作上有较大突破，全市实际利用外资和引进市外资金有较大增长。精心搞好第23届牡丹花会招商引资活动。认真抓好已签约重大项目的跟踪落实，切实提高资金到位率和项目开工率。完善鼓励外贸出口的政策支持，大力调整出口商品结构，巩固传统大宗产品出口，积极培育新的出口增长点，突出抓好机电产品和高新技术产品的出口，力争全年外贸出口6亿美元以上。

　　进一步加快非公有制经济发展。完善和落实相关政策，鼓励非公有资本进入法律法规没有禁止的一切行业和领域，支持非公有制资本参与国有企业改制和重组，努力为非公有制企业创造平等竞争的发展环境。继续实施“四个一批”和“骨干带动工程”，催生一批技术含量高、市场竞争力强的优势企业。力争营业收入超亿元的非公有制企业达到65家，其中北方易初、兴安线材、银辉冶炼、伟业轧钢、洛钢孟津第一轧钢厂等5家力争超10亿元。

　　发展壮大县域经济。认真落实支持县域经济发展的各项政策，积极争取省政府县域经济发展专项资金，加快县城环保、旅游等基础设施建设。坚持以招商引资、发展非公有制经济和现有企业改制为突破口，大力发展骨干企业和特色产业。偃师、新安、伊川等县(市)要进一步瞄准先进，争取进入全省乃至全国先进行列；其它各县要坚定信心，打好基础，积极培育支柱产业，努力提高在全省的位次；各城市区要结合旧城改造和新区开发，加快发展第三产业，进一步繁荣城区经济。市政府各部门都要进一步加大对县域经济发展的支持力度，努力提供优质服务。

　　(六)加强财政金融工作，更好地支持经济发展

　　依法强化财政收支管理。加大税收征管力度，堵塞各种漏洞，努力做到应收尽收。严肃税收纪律，任何地方和部门不得越权减免税或擅自出台税收优惠政策。加强新增税源监控，狠抓契税等小税种的征管。加强国有资产管理，确保国有资产保值增值，确保国有资本投资收益。严格执行行政性收费“收支两条线”规定，切实做到应收尽收，未经市政府批准，任何单位和个人不得减免。全面推进部门预算、国库集中支付和政府采购等改革。优化财政支出结构，严格控制一般性支出，确保社会保障等重点支出。科技、教育、农业等专项资金要科学使用，集中解决重点问题。继续抓好清理小金库工作。进一步加大对重点部门、重点资金、重点项目的审计监督力度。

　　充分发挥金融对经济增长的支持作用。加强银企沟通，搞好项目对接，引导金融机构积极支持符合国家产业政策的项目，尽快落实银企洽谈会签约资金。各级政府和有关部门要积极帮助银行加大贷款回收力度，严厉打击恶意逃废银行债务行为，构建良好金融生态环境。全面深化农村信用社改革，加大清收不良贷款力度，确保专项央行票据如期兑付。完善基层农村信用社法人治理结构，建立现代企业制度，创新经营机制，健全内控制度，使其成为支持“三农”的主力军。

　　(七)加强环境和资源保护，统筹发展各项社会事业

　　大力实施“碧水蓝天”工程。加快创建国家环保模范城市步伐，市财政预算5000万元用于环保工作，有关部门要严格资金管理，确保真正发挥效益。突出抓好大气污染治理，彻底取缔燃煤小锅炉，杜绝拖拉机进城，坚决关停“十五小”企业，加强超标排污企业治理，加快重污染企业搬迁，严禁在城市规划区新上污染项目。进一步完善环境保护目标责任制，坚持重大环境违法案件专人负责、限时办理制度。争取全年城市空气质量良好天数达到220天以上，确保伊洛河出境断面水质达到省定标准。

　　推进生态城市建设。加强龙门山、周山、小浪底、上清宫4大森林公园的建设管理，进一步完善功能，提高品位。加快洛河南岸隋唐城遗址7平方公里的农业科技园区建设。抓好洛河两岸生态景观带建设，完善洛浦公园堤内设施和水面景观。加快环保基础设施建设，确保涧河、瀍东、新区污水处理厂和偃师、新安、嵩县县城污水处理厂如期竣工。积极做好其它县城污水处理厂和垃圾无害化处理场前期工作，力争早日开工建设。

　　加强资源保护和有序开发。坚持可持续发展战略，引导全社会树立节约能源、节约资源的意识，努力建设节约型城市。以推进资源节约和综合利用为重点，大力发展循环经济。坚持政府推进与市场运作相结合，加大钼、煤、铝矾土等矿产资源整合力度，优化资源配置，严禁乱采乱挖，确保矿产资源整合工作取得新成效。合理开发和节约使用水、土地等资源，提高资源利用效益。

　　认真落实科教兴洛战略。进一步加大科技投入，加强科技攻关，发展壮大高新技术产业。加快科研院所企业化转制和产权制度改革，推进产学研结合，促进科研成果就地转化，力争原部属的12家科研院所技工贸总收入达到75亿元。加快企业技术中心建设，推动企业成为技术创新主体。加快省、部共建洛阳装备制造业基地步伐，推进制造业信息化示范项目建设，不断提高“洛阳制造”的核心竞争力。进一步提升高新区的创新能力和经济总量，加快火炬创新创业园建设，确保引进超千万元项目15个以上。巩固提高教育“两基”成果，积极发展高中阶段教育，初中毕业生升入高中阶段比例达到60%以上。整合优化职业教育资源，加快重点职业技术学校基础设施建设。大力发展高等教育，不断扩大招生规模。引导大专院校围绕洛阳经济发展，加强重点学科和重点实验室建设，突出抓好河南科技大学先进制造技术实验室建设。优化资源配置，鼓励企业和高校共建实验室。继续做好洛阳大学、洛阳工业高专升本科工作。加强人才队伍建设，进一步完善留住骨干科技人才的政策措施，积极培养和引进科技拔尖人才、专业技术人才和高技能劳动者。

　　大力发展各项社会事业。加快城乡公共卫生体系建设，健全卫生监督、疾病控制、紧急救援和妇幼保健体系。进一步整合医疗资源，加强重大疾病的预防和控制，提高突发公共卫生事件的快速处理能力。全面建成疾病预防控制中心，加快正骨医院高等级病房建设。大力发展社区卫生服务，巩固新型农村合作医疗试点工作。积极扶持中医、中药事业发展。加大投入，完善机制，努力创建国家卫生城市。加强文化基础设施建设，积极开发利用河洛文化资源，繁荣广播电视和文化艺术，丰富群众文化生活，培育健康有序的文化市场。做好历史文化名城和文物保护利用工作。落实人口和计划生育目标责任制，稳定低生育水平。广泛开展全民健身活动，提高竞技体育水平。推进统计改革和建设，不断提高统计数据的准确性。认真做好第一次全国经济普查工作。继续做好民族宗教、外事、人防、气象、地方志等工作。

　　(八)坚持以人为本，努力构建和谐洛阳

　　切实加强就业和社会保障工作。全面落实财政投入、小额贷款、免费培训、税费减免等支持就业再就业的各项政策，坚持在加快发展中扩大就业，力争城镇新增就业8万人，其中下岗失业人员再就业4万人。完善公共就业服务体系，积极开展再就业援助，重点帮助“4050”人员等困难群体就业。进一步巩固“两个确保”，依法扩大社会保障覆盖面，加大社会保险征缴和清欠力度，力争养老保险参保人数新增2万人以上，清欠8000万元。做好下岗职工出中心工作，实现国有企业下岗职工基本生活保障向失业保险并轨。加快“金保工程”建设，促进五险合并征缴，实行养老保险市级统筹。严格各类社会保险资金管理，确保基金安全。

　　认真解决关系人民群众切身利益的问题。努力增加城乡居民收入。加大清理拖欠工程款和农民工工资力度，年内力争完成清欠90%以上，严禁发生新的拖欠。建立健全城乡社会救助体系，进一步规范城市“低保”，加强农村特困户救助，完善农村“五保户”生活保障制度。关心弱势群体，做好农村受灾群众生活救济工作，确保困难群众有饭吃、有衣穿、有房住、有病能医、不让一个孩子因家庭贫困而失学。切实做好扶贫开发工作，加大农户小额贷款贴息和农民培训力度，积极实施移民式扶贫。加大整顿和规范市场经济秩序力度。搞好农业生产资料供应和价格监管，坚决打击坑农害农行为。严厉打击制售假冒伪劣食品、药品等违法犯罪活动，保护人民群众身体健康和生命安全。

　　全力维护社会稳定。加强社会治安综合治理，推进社会治安防控体系建设，争创“全国综治先进市”。坚持严打方针，严厉惩处各种违法犯罪活动，维护正常的社会秩序。高度重视安全生产，认真落实责任追究制度，强化技术、投入等关键措施，全面排查和治理事故隐患，坚决遏制重特大安全事故的发生。切实做好人民群众来信来访工作，坚持经常性矛盾纠纷排查调处，努力把各种矛盾和问题解决在基层、化解在萌芽状态。积极预防和妥善处置群体性事件，确保社会大局稳定。

　　(九)搞好精神文明建设，推进民主法制进程

　　加强精神文明建设。全面落实公民道德建设实施纲要，深入开展社会公德、职业道德和家庭美德教育，加强未成年人思想道德建设，努力在全社会形成良好道德风尚。广泛开展群众性精神文明创建活动，抓好文明景区、文明城市创建工作。加强诚信体系建设，倡导诚实守信的良好社会风气。积极开展法律、科技、文体、卫生进社区和文化、科技、卫生“三下乡”活动。加强国防教育，关心支持驻洛解放军、武警部队和民兵预备役建设，增进军政军民团结，力夺全省双拥模范城五连冠。

　　加强民主法制建设。坚持依法治市，加强法制宣传教育，提高全民法律素质。严格执行人大及其常委会通过的各项法规、决定和决议，自觉接受其工作监督和法律监督，支持人大代表依法行使职权。加强与人民政协的协商，接受人民政协的民主监督。认真办理人大代表议案、建议和政协委员提案。广泛听取各民主党派、工商联的意见，发挥工会、共青团、妇联等人民团体的桥梁纽带作用。依法保护妇女儿童、残疾人和未成年人合法权益。积极开展法律援助，依法保障弱势群体的合法权益。巩固和扩大基层民主，完善村民自治、社区民主管理和企业民主管理制度，做好第五届村民委员会换届选举工作。

　　(十)加强政府自身建设，努力提高政府工作水平

　　进一步转变政府职能。大力推进政企分开、政资分开、政事分开。在继续履行好政府经济调节、市场监管职能的同时，进一步强化社会管理和公共服务职能，真正把政府职能转到为市场主体服务和创造良好发展环境上来，把更多的精力放到推进社会发展和解决民生问题上来，努力建设服务型政府。进一步改进服务方式，提高工作效率和服务质量。积极推进电子政务，完善行政服务大厅功能，真正做到便民高效。坚持政务公开，增强政府工作透明度。

　　全面推进依法行政。深入贯彻行政许可法和全面推进依法行政实施纲要，积极建设法治政府。进一步做好政府立法和行政复议工作。加强和改进行政执法，努力做到严格、公正、文明执法。坚持行政执法责任制和执法过错追究制，认真解决有法不依、执法不公和多头执法等群众反映强烈的问题。加强对行政权力的监督，规范行政行为，依法履行职责，做到有权必有责、用权受监督、侵权要赔偿、违法要追究。

　　努力提高经济社会管理水平。进一步改进经济管理的方式方法，自觉按市场规律办事，努力提高驾驭社会主义市场经济的能力。处理好速度与质量、结构、效益之间的关系，切实把工作着力点放在提高经济增长的质量和效益上。立足当前，谋划长远，认真做好“十一五”规划编制工作。充分发扬民主，善于集中民智，推进决策民主化和科学化。进一步改进社会管理，依法管理和规范社会组织、社会事务，协调处理好各种利益关系。

　　切实加强作风建设。牢固树立科学发展观和正确政绩观，坚持以人为本、执政为民，始终做到人民利益高于一切，多做打基础、管长远、惠及百姓的实事。以保持共产党员先进性教育活动为契机，切实转变政府机关工作作风。加强公务员队伍建设，进一步提高公务员综合素质，严格执行《洛阳市国家行政机关及其工作人员行政过错责任追究暂行办法》。规范各类检查评比活动，除安全生产、环保、税务等国家法律规定的检查外，各部门原则上不得组织检查评比，确需进行的，必须报经市政府批准。大兴求真务实之风，大力精简会议、文件，坚决改进会风、文风，切实减少应酬和不必要的事务性活动。强化责任意识，狠抓工作落实，各项工作都要细化量化，责任到人，奖惩到位，确保如期完成。牢记“两个务必”，始终保持谦虚谨慎、不骄不躁、艰苦奋斗的优良作风。坚持廉洁从政、从严治政，旗帜鲜明地反对、预防和惩治腐败。

　　各位代表，全面建设小康社会，再创洛阳新的辉煌，是全市人民的共同心愿和奋斗目标。让我们以邓小平理论和“三个代表”重要思想为指导，抓住机遇，乘势而上，锐意进取，扎实工作，为超额完成“千百亿”目标、全面建设小康社会而努力奋斗！
